# Usefulness of *Plasmodium falciparum*-specific rapid diagnostic tests for assessment of parasite clearance and detection of recurrent infections after artemisinin-based combination therapy

**DOI:** 10.1186/1475-2875-12-349

**Published:** 2013-10-01

**Authors:** Berit Aydin-Schmidt, Marycelina Mubi, Ulrika Morris, Max Petzold, Billy E Ngasala, Zul Premji, Anders Björkman, Andreas Mårtensson

**Affiliations:** 1 Malaria Research, Department of Medicine-Solna, Karolinska University Hospital/Karolinska Institutet, Stockholm, Sweden; 2 Muhimbili University of Health and Allied Science, Dar es Salaam, Tanzania; 3 Division of Global Health, Department of Public Health Sciences, Karolinska Institutet, Stockholm, Sweden; 4 Centre for Applied Biostatistics, Sahlgrenska Academy, University of Gothenburg, Gothenburg, Sweden

**Keywords:** Malaria, RDT, LDH, HRP2, Clearance, Recurrent parasitaemia, Treatment follow-up

## Abstract

**Background:**

Rapid diagnostic test (RDT) is an important tool for parasite-based malaria diagnosis. High specificity of RDTs to distinguish an active *Plasmodium falciparum* infection from residual antigens from a previous infection is crucial in endemic areas where residents are repeatedly exposed to malaria. The efficiency of two RDTs based on histidine-rich protein 2 (HRP2) and lactate dehydrogenase (LDH) antigens were studied and compared with two microscopy techniques (Giemsa and acridine orange-stained blood smears) and real-time polymerase chain reaction (PCR) for assessment of initial clearance and detection of recurrent *P. falciparum* infections after artemisinin-based combination therapy (ACT) in a moderately high endemic area of rural Tanzania.

**Methods:**

In this exploratory study 53 children < five years with uncomplicated *P. falciparum* malaria infection were followed up on nine occasions, i.e., day 1, 2, 3, 7, 14, 21, 28, 35 and 42, after initiation of artemether-lumefantrine treatment. At each visit capillary blood samples was collected for the HRP2 and LDH-based RDTs, Giemsa and acridine orange-stained blood smears for microscopy and real-time PCR. Assessment of clearance times and detection of recurrent *P. falciparum* infections were done for all diagnostic methods.

**Results:**

The median clearance times were 28 (range seven to >42) and seven (two to 14) days for HRP2 and LDH-based RDTs, two (one to seven) and two (one to 14) days for Giemsa and acridine orange-stained blood smear and two (one to 28) days for real-time PCR. RDT specificity against Giemsa-stained blood smear microscopy was 21% for HRP2 on day 14, reaching 87% on day 42, and ≥96% from day 14 to 42 for LDH. There was no significant correlation between parasite density at enrolment and duration of HRP2 positivity (r = 0.13, p = 0.34). Recurrent malaria infections occurred in ten (19%) children. The HRP2 and LDH-based RDTs did not detect eight and two of the recurrent infections, respectively.

**Conclusion:**

The LDH-based RDT was superior to HRP2-based for monitoring of treatment outcome and detection of recurrent infections after ACT in this moderately high transmission setting. The results may have implications for the choice of RDT devices in similar transmission settings for improved malaria case management.

**Trial registration:**

Clinicaltrials.gov, NCT01843764

## Background

Parasite-based *Plasmodium falciparum* diagnosis is generally recommended by the World Health Organization (WHO) to target artemisinin-based combination therapy (ACT) to patients with confirmed malaria infections [1]. This is important in order to prevent overuse of ACT, reduce costs, minimize development and spread of anti-malarial drug resistance and to improve management of other causes of fever [2,3]. The availability of malaria rapid diagnostic tests (RDTs) constitutes an opportunity for parasite-based malaria diagnosis in rural African settings beyond the reach of microscopy services [4].

Malaria RDTs are based on detection of parasite antigens. The main antigen targeted is histidine-rich protein 2 (HRP2), which has proven to be a highly sensitive and stable marker for identification of *P. falciparum* infection. However, a concern with HRP2-based RDTs is the presence of residual antigenaemia resulting in persistent positive test results during several weeks after a successful treatment [5,6]. This is of particular concern in moderate/high transmission areas where false positive RDTs may frequently result in provision of anti-malarial treatment to patients who are not malaria infected. This phenomenon may also impair health workers trust in and adherence to RDT results [7,8].

Another antigen used in RDTs is parasite-specific lactate dehydrogenase (LDH), either species specific, i.e., *P. falciparum* LDH (*Pf*LDH) and *Plasmodium vivax* (*Pv*LDH), or pan-*Plasmodium* (pLDH), detecting all five human malaria species [9]. LDH-based RDTs have generally been shown to be less heat stable and sensitive than HRP2-based RDTs for detection of *P. falciparum*, but they are more specific since LDH is rapidly cleared from the blood following a successful anti-malarial treatment. Consequently, LDH-based RDTs do not remain positive after parasite clearance [2,10]. Interestingly, some recently available *Pf*LDH-based RDTs have shown sensitivities and heat stabilities similar to HRP2-based RDTs [11-14].

Previous RDT studies in this particular field have mainly focused on post-treatment clearance and have primarily followed patients until the tests have become negative [12,13]. Thus, insufficient data are available on the efficiency of RDTs to identify recurrent infections after recently cleared infections in moderate/high transmission areas. This may be of particular importance in an era of increasing drug resistant malaria [15]. The aim of this study was therefore to investigate clearance and detection of recurrent *P. falciparum* infections of HRP2 and LDH-based RDTs during 42 days after initiation of artemether-lumefantrine treatment in children with uncomplicated malaria in a moderately high endemic area of rural Tanzania. Furthermore, since no previous study has included clearance by polymerase chain reaction (PCR) as a comparator, this was done to allow a more comprehensive evaluation of RDT for monitoring of anti-malarial treatment outcome. The entire assessment herein reported thus included two RDTs (HRP2 and LDH-based) for antigen detection, compared with two microscopy techniques (Giemsa-stained thick blood smears and acridine orange-stained thin blood smears) for whole parasite detection and real-time PCR for detection of parasite DNA.

## Methods

### Study site and population

This health facility-based study was conducted during the peak seasons for malaria transmission, in June to September 2009 and July to October 2010 at Mlandizi health centre, Kibaha district and during March to May 2011 in Fukayosi dispensary, Bagamoyo district, both located in Coast Region, Tanzania. Artemether-lumefantrine was introduced as first-line treatment for uncomplicated malaria in 2006 in the study area, whereas RDT had not yet been implemented for parasite-based malaria diagnosis. At the time of the trial malaria transmission was considered to be moderately high in both study sites.

The Mlandizi health centre provides basic in- and outpatient care for a population of approximately 33,000. Laboratory services, including malaria microscopy, are available during office hours. The monthly blood smear positivity rate among febrile children < five years of age was 33% (range 20-48%) and 15% (range 11-21%) during the sampling period in 2009 and 2010, respectively. There was a period of artemether-lumefantrine stock-out in Mlandizi in 2009. During this period patient recruitment was stopped. There was an on-going insecticide-treated bed net campaign in the area markedly reducing the incidence of malaria among children < five years between 2009 and 2010.

Fukayosi dispensary provides basic outpatient care for a population of about 7,000. Malaria microscopy service is available seven days a week. Malaria blood smear positivity rate was 19% (74/384) among febrile children < five years of age during the conduct of the trial.

Participating laboratory staff and study nurses at the two study sites received one day’s training in performance and interpretation of both RDTs before the start of the study.

### Study design and sample collection

Children between six and 59 months presenting at the study sites with fever, i.e., measured axillary temperature of ≥37.5°C or a history of fever during the preceding 24 hours, and a positive screening blood slide for *P. falciparum* mono-infection with a parasite density of 2,000-250,000/μL, and willing/able to comply with the 42 days follow-up were eligible to participate in the study. Children with a history of anti-malarial drug intake within two weeks or symptoms/signs of severe disease were excluded. Written informed consent was obtained from a parent/guardian of all enrolled children.

At enrolment, i.e., day 0, a complementary finger-prick capillary blood sample was taken for two thick and thin smears, and two RDTs. In addition, approximately 50 μL of blood was spotted on a filter paper. All enrolled children were treated with artemether-lumefantrine (Coartem®) in standard doses based on body weight, according to national treatment guidelines [16]. Only the initial drug dose was given under supervision. Enrolled children were requested to return for clinical review and blood sampling on days 1, 2, 3, 7, 14, 21, 28, 35 and 42, or anytime if condition deteriorated or fever re-occurred. At each follow-up visit all day 0 blood tests were repeated, except for the Giemsa-stained thin smear, which was used for confirmation of *P. falciparum* mono-infection solely on day 0. A case record form was completed at enrolment by a clinical officer, with clinical and demographic information, including age, sex and information on use of insecticide-treated bed nets. Body temperature, symptoms and prescription of drugs were recorded in the case record forms on all visits.

Whenever fever and/or any other symptoms/signs of disease re-occurred during follow-up, a Giemsa-stained blood slide was to be read directly at the health centre and if positive for *P. falciparum* the child was retreated with artemether-lumefantrine.

The only incentive given to the study participants was bus fares to cover travel costs during follow up visits.

### Laboratory procedures

#### Rapid diagnostic tests

Two RDTs, ParaHIT ®*f* (Span Diagnostics Ltd, Surat, India), detecting *P. falciparum*-specific HRP2 antigen (hereafter referred to as HRP2) and CareStart™ Malaria (G0151), (Access Bio, Inc, NJ, USA), detecting *P. falciparum*-specific LDH antigen (hereafter referred to as LDH), were performed and interpreted on site according to the manufacturer’s instructions. ParaHit was, at the time of the study, approved by the Tanzanian National Malaria Control Programme and was the most deployed RDT. The single *Pf* CareStart test was chosen based on the heat stability and performance of the CareStart pan-LDH test in the WHO product testing 2009, where it was among the best performing LDH-based tests for *P. falciparum* detection [17]. Both RDTs are two band tests, i.e., one test band specific for *P. falciparum* and one control band.

A laboratory technician or study nurse, blinded to any Giemsa-stained blood smear result, performed, interpreted and recorded the RDT results. Very faint bands at the test line position were to be defined as positive. Band intensity was not recorded. In case the control line did not appear, the result was considered invalid and the test was repeated. The RDT kits were stored at <30°C prior to use, the temperature being recorded daily.

#### Giemsa-stained blood smear microscopy

One of the two thin blood smears collected on day 0 and the thick blood smears from all sampling points were stained with 5% Giemsa for 20 minutes at the health facilities, after which they were transported once weekly to Muhimbili University of Health and Allied Sciences (MUHAS) in Dar es Salaam. The day 0 Giemsa-stained thin smear was examined for confirmation of *P. falciparum* mono-infection. One of the two thick smears was examined by two independent, experienced microscopists, who were unaware of the RDT results, at MUHAS. A total of 200 microscopic fields (×100 magnification) were examined before a smear was considered negative. Asexual parasite densities were calculated by counting parasites against 200 white blood cells (WBC), assuming 8,000 WBC/μL of blood. If less than 10 parasites were detected per 200 WBC, estimates were made against 500 WBC [18]. Gametocytaemia was assessed by reading 200 microscopic fields. All blood slides with discrepant results, defined as >50% difference in parasite density or a positive *versus* negative result between the readers, were subjected to a third blinded reading at Karolinska Institutet (KI), Sweden. In addition, all blood slides from children showing a negative thick blood smear and a positive PCR at the same time point, as well as a random sample of 10% of all blood slides, plus the blood slides from the time point of each participant with the last CareStart positive, and the first CareStart negative results, were subjected to microscopy reading at KI for quality control. The mean of the two most concordant counts were used to calculate the final parasite density [18]. In case of discrepancy regarding positive *versus* negative results between the first two readers and the third, the third reading at KI was defined as decisive.

#### Acridine orange blood slide microscopy

The thin blood smears from all sampling points were subjected to acridine orange staining and reading at Muhimbili University Hospital. The thin smears were fixed in methanol. A solution of 0.01% acridine orange in phosphate buffer (pH 7.2) with 5% glycerine was then applied to the smears, which were read in a fluorescence microscope at ×40 magnification [19]. The results were recorded as either positive or negative, i.e., parasite counts were not assessed. The microscopists were unaware of all previous RDT and microscopy results.

#### DNA detection by PCR

Approximately 50 μL of blood seeded on a filter paper (Whatman 3MM®) from all sampling points were collected for molecular analysis. The filter papers were dried and put in individual plastic bags and transported to KI. The filter papers were stored at <30°C until processed. Three 3-mm punches (approximately 10–15 μL) from each filter paper sample were extracted with a modified version of the ABI 6100 Nucleic Acid Prep Station protocol (Applied Biosystems, USA) as previously described [20]. DNA was eluted in 200 μL of buffer. All samples were analysed for presence of *Plasmodium* DNA by an 18S rDNA probe based real-time PCR assay [21]. A cut-off value for positivity was set at a cycle threshold (Ct) of <40. All samples were run in triplicates. Samples where one out of three had a Ct-value <40 were repeated in triplicates with the same real-time PCR. Samples with repeated single Ct-values <40 or a Ct average of >38.5 were subjected to a confirmatory/decisive *P. falciparum*-specific nested PCR [22]. Two positive *P. falciparum* controls (5, 10 or 50 parasites/μL) as well as negative controls were included in each 96-well PCR plate.

#### PCR genotyping to distinguish re-infections from recrudescence

Filter-paper blood spots from patients with recurrent PCR positivity during follow-up were re-extracted using the Chelex-100 method [23]. Stepwise genotyping with three highly polymorphic genetic markers, i.e., the *merozoite surface protein* (*msp*) 1, *msp* 2 and *glutamate rich protein* (*glurp*) was performed according to standard protocols to differentiate re-infection (new infection) from recrudescence (treatment failure). For each marker, recrudescence was defined as the presence of at least one matching allelic band and re-infections were defined as the absence of any matching allelic band in samples at enrolment (day 0) and at day of recurrent infection [24,25].

#### Definition of clearance time and recurrent infections

Clearance time was defined as the first sampling day after initiation of treatment when a test result was negative. There were two exceptions to this. First, when a RDT result turned negative for one sampling day, followed by a positive RDT result again the following sampling day, the negative result was ignored if the PCR and/or BS results did not indicate presence of a recurrent infection. Second, when one negative PCR result was followed by a positive PCR result the following sampling day up to day 7, the negative result was ignored. From day 14 and onwards the clearance time was calculated from the first day with negative result.

Recurrent infection was defined as detection of *P. falciparum* DNA (PCR) confirmed by a positive Giemsa-stained blood smear microscopy and/or LDH during follow-up after the initial infection had been cleared.

#### Study outcomes and statistical analysis

The primary outcomes were clearance time and detection of recurrent infection with the five diagnostic tests. Secondary outcomes included specificity of the two RDTs against Giemsa-stained blood smear microscopy (gold standard), identification of PCR-adjusted re-infection/recrudescence among the recurrent infections and correlation between parasite density at enrolment and persistence of HRP2. In the calculation of correlation between day 0 parasite densities and duration of HRP2 positivity, seven children were included who had cleared HRP2 positivity before they were lost to follow-up. These seven children were not included in any other analysis.

The study was considered exploratory, which precludes a power calculation. A sample of ≥50 children was predefined.

Data were entered in Microsoft Excel® and analysed using STATA 12® software. Categorical variables were compared using Fisher’s exact test. Pearson linear correlations were calculated in SPSS. Sensitivity and specificity of acridine orange against Giemsa-stained blood smear microscopy (gold standard) and both microscopic methods against PCR (gold standard) was calculated. Statistical significance was stated at the 5% level and 95% confidence intervals (CI) are presented.

### Ethical considerations

The study was conducted in accordance with the Declaration of Helsinki and Good Clinical and Laboratory Practices. It was approved by the Directorate of Research and Publications, MUHAS (Ref.No.MU//RP/AEC/Vol.XIII/142) and the Regional Ethics Committee, Stockholm, Sweden.

The study is registered at http://www.clinicaltrials.gov with study identifier NCT01843764.

## Results

### Study subjects

**Figure 1:**
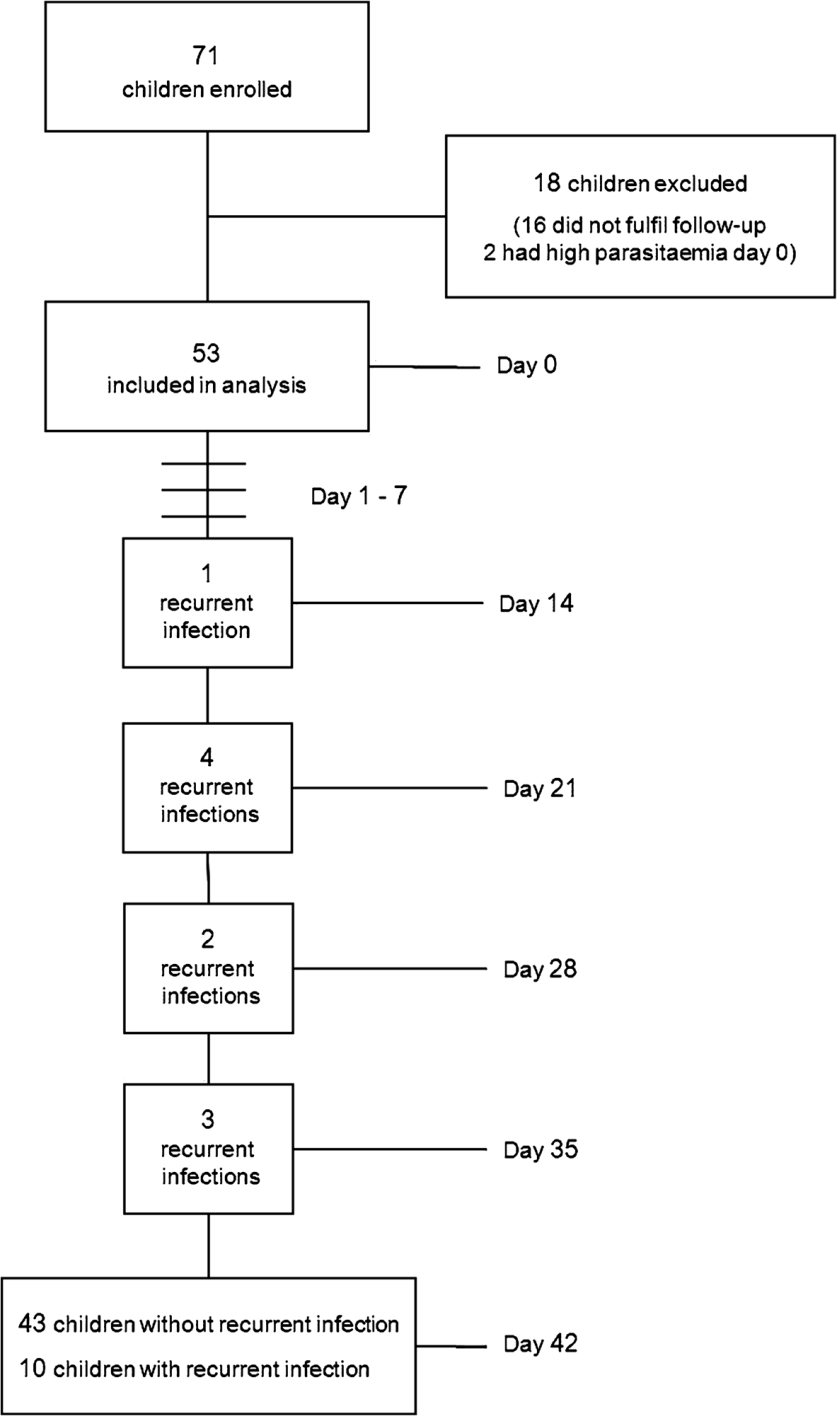
Flow of patients through the study.

The flow of patients through the trial is outlined in Figure 1. Two children were excluded from the analysis due to high parasite densities at enrolment, i.e., 992,000 and 494,400/μL. Another 16 children were not able to fulfil the stipulated follow-up, the most common reason being leaving the study area or long distance to the health centre.

All children were positive with both RDTs at day of inclusion. Baseline characteristics of the 53 children included in the analysis are presented in Table 1.

**Table 1:** Baseline characteristics of the 53 children included in the analysis

Age in months, mean	42 (range 10–59)
Sex male/female	35/18
Axillary temperature °C, mean	37.7 (range 37.0-39.0)
Axillary temperature ≥37.5°C	83% (44/53)
Duration of fever, mean days	2.5 (range 1–4)
Other complaints*	64% (34/53)
Geometric mean parasite density /μL	37,640 (range 2,000-250,000)
Sleeping under insecticide-treated bed net	85% (45/53)

*vomiting n = 18.

abdominal pain n = 9.

cough n = 11.

diarrhoea n = 3.

### Clearance time

**Figure 2:**
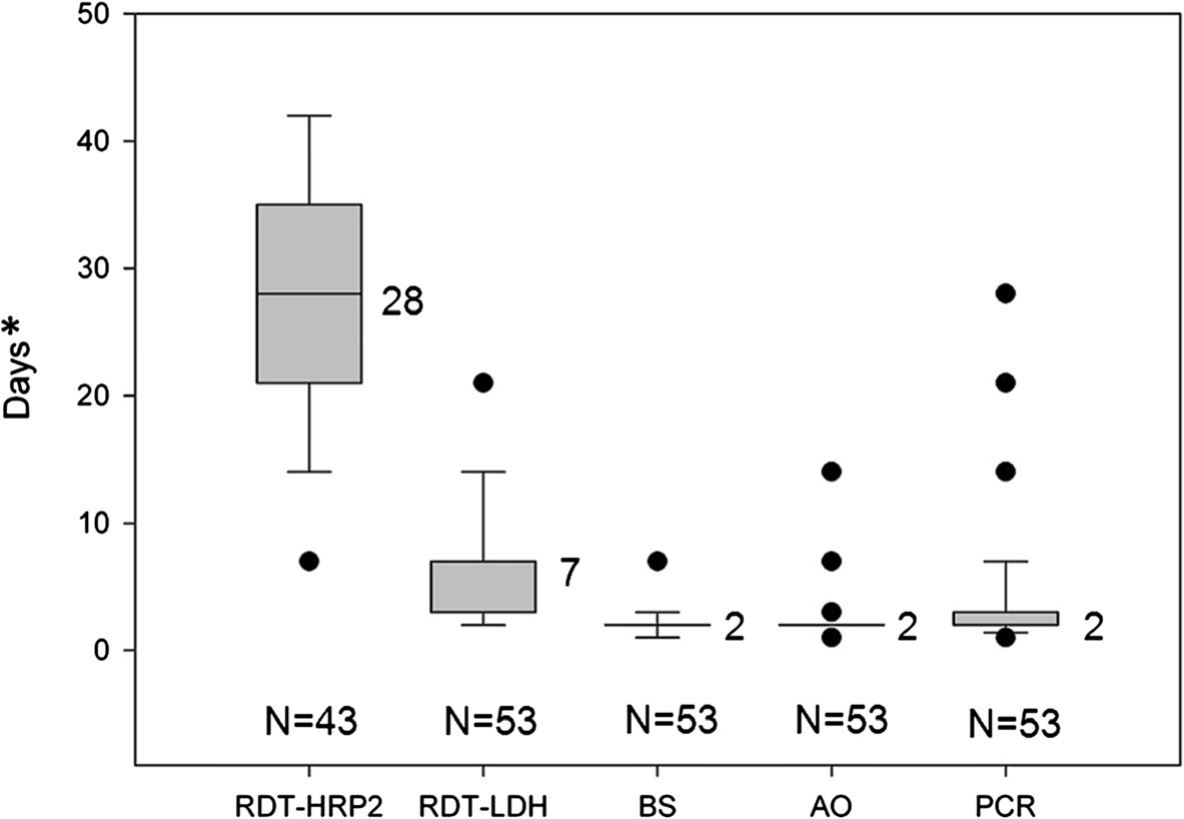
**Median clearance times for the five diagnostic tests.** N = number of patients included in the analysis. BS = Giemsa-stained blood smear. AO = acridine orange-stained blood smear. * Last day of follow-up = day 42.

**Figure 3:**
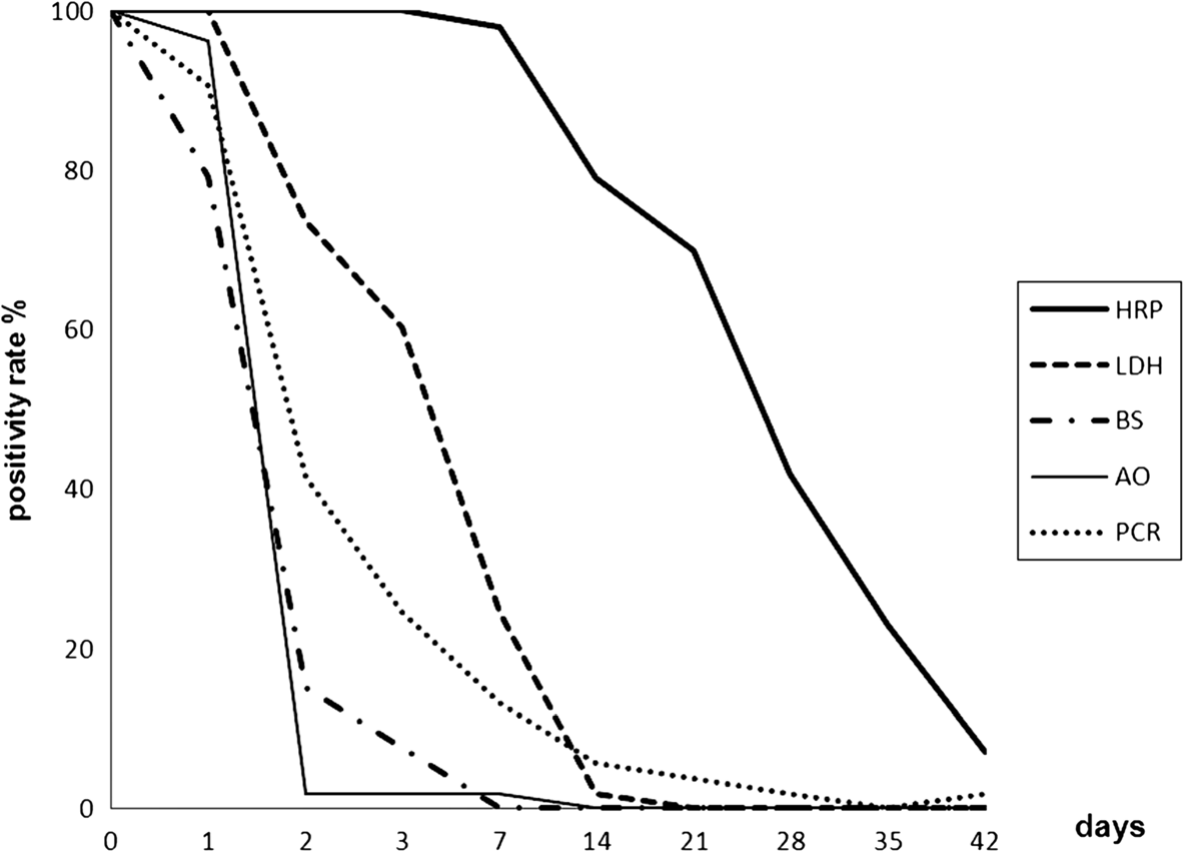
**Positivity rates at each sampling point by the five diagnostic tests.** HRP2 data represent 43 patients and the remaining diagnostic tests 53 patients. HRP = HRP2 based RDT. LDH = LDH based RDT. BS = Giemsa-stained blood smear. AO = acridine orange-stained blood smear. PCR = real-time PCR.

The calculations for HRP2 clearance are based on the 43 children without recurrent infection during follow-up, since eight of the ten children with parasite recurrence remained HRP2-positive from enrolment up to the time of recurrent infection. All other tests, i.e., LDH, Giemsa and acridine orange-stained blood smears as well as PCR cleared before parasite recurrence. Consequently, clearance calculations for these tests are based on data from all 53 children. The median clearance times for the five diagnostic tests are presented in Figure 2 and their respective positivity rates at each sampling point are shown in Figure 3.

**Figure 4:**
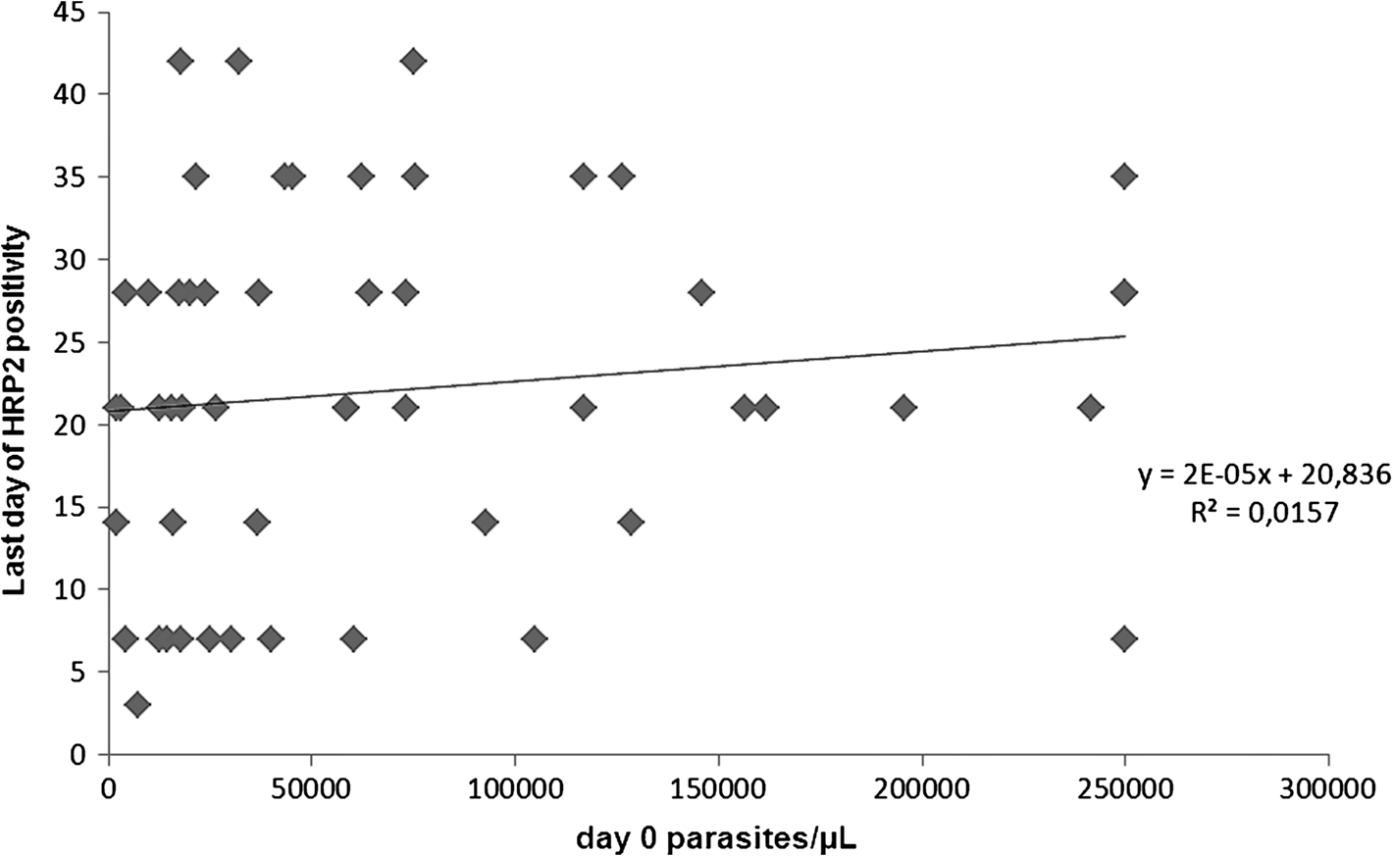
**Correlation between parasite density at enrolment and duration of HRP2 positivity.** N = 52, including 43 children without re-infection, two with cleared positivity before re-infection and seven with cleared positivity before being lost to follow-up. Correlation coefficient (r) = 0.13 (p = 0.38).

The geometric mean clearance time for HRP2 was 26.4 (95% CI 23.0-30.3) days. One patient cleared HRP2 by day 7 and three remained positive up to day 42 (last day of follow-up). The false positivity rates for HRP2 against PCR on days 14, 21, 28, 35 and 42 were 80% (32/40), 64% (27/42), 43% (18/42), 24% (10/42) and 7% (3/41), respectively. All PCR positive results were also HRP2-positive throughout the study. There was no significant correlation between parasite density at enrolment and duration of HRP2 positivity (r = 0.13, p = 0.38) (Figure 4). For LDH the mean clearance time was 5.5 days (95% CI 4.5-6.7). No significant correlation between parasite density at enrolment and duration of LDH positivity was observed (r = 0.21, p = 0.11).

The mean clearance time for Giemsa-stained blood smears was 2.0 days (95% CI 1.8-2.3). Four children (8%) remained positive by microscopy until day 3. Two children were microscopy positive day 2 and 3, respectively, with parasite densities of 16 and 32/μL each. However, PCR was negative for both samples at these time points. There was no significant association between clearance times of Giemsa-stained blood smears and HRP2 (p = 0.50).

Acridine orange-stained blood smears had a mean clearance time of 2.1 days (95% CI 1.9-2.3). The mean clearance time for PCR was 2.9 days (95% CI 2.3-3.6). Persistent PCR positivity up to day 7, 14 and 21 was observed in one, one and two children, respectively. In two of these four children, solely gametocytes were detected by Giemsa-stained blood smear microscopy at two and three sampling points, respectively, during the time of persistent PCR positivity.

### Recurrent infections

**Figure 5:**
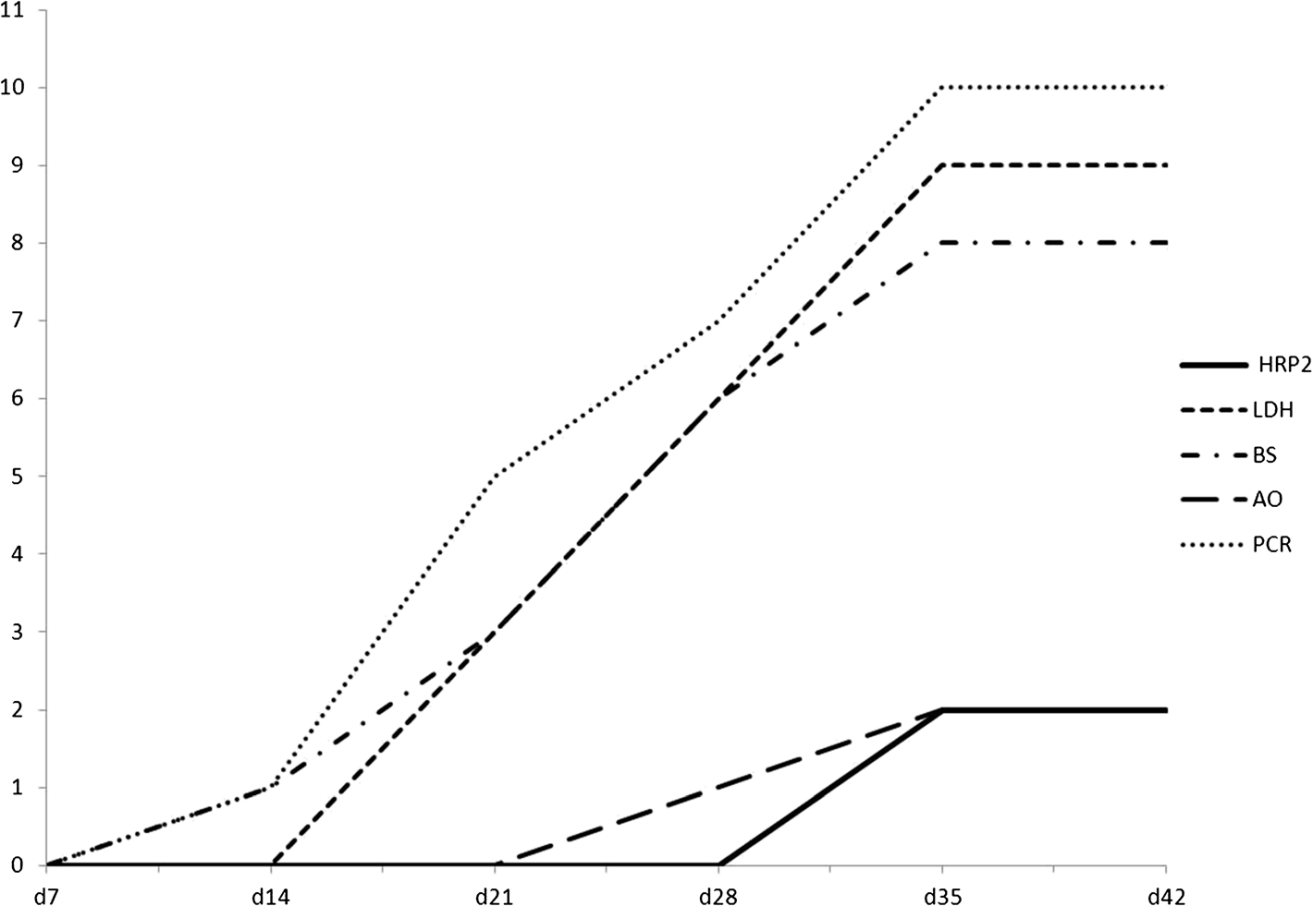
**Cumulative positivity by the diagnostic tests for the ten recurrent *Plasmodium falciparum *infections detected during follow-up.** BS = Giemsa-stained blood smear. AO = acridine orange-stained blood smear.

Ten children had recurrent *P. falciparum* infection during the 42 day follow-up as assessed by PCR, Giemsa-stained blood smear and/or LDH. One recurrent infection was defined as recrudescence, six as re-infections, whereas three were undetermined by PCR genotyping (Table 2). Eight of these ten children had remaining HRP2 positivity from the initial infection. Thus, only two recurrent infections, both occurred on day 35, were detected by HRP2. Conversely, LDH detected eight (80%) recurrent infections at the day of parasite recurrence. Furthermore, one additional child had a positive LDH result on the following visit. Giemsa-stained blood smear microscopy identified eight of the ten recurrent infections, whereas acridine orange detected one. PCR identified all ten infections at the time of parasite recurrence. However, six more patients experienced a single occasion of PCR positivity during follow-up between days 21 and 42. All these six children were asymptomatic by the time of the transient PCR positivity. Except for two patients who were still HRP2-positive, all other tests were negative at the time of PCR positivity. Thus, none of the six children fulfilled the definition of recurrent infection. Cumulative positivity by the respective diagnostic tests for the ten recurrent infections detected during follow-up is shown in Figure 5.

**Table 2:** **Characterization of the ten recurrent *Plasmodium falciparum *infections detected during follow-up**

	**Diagnosis day**	**Symptom**	**Diagnostic tool**			**Parasites**	**New infection/**
			**PCR**	**Giemsa**	**LDH-RDT**	**/μL**	**Recrudescence**^**2**^
1	14	A	+	+		80	New
2	21	A	+	+	+	1,600	New
3	21	R	+		+		undertermined
4	21	^1^	+	+	^1^	600	recrudescence
5	21	F	+		+		undertermined
6	28	A	+	+	+	316,000	new
7	28	F	+	+	+	229,600	new
8	35	F	+	+	+	61,240	undertermined
9	35	F	+	+	+	9,800	new
10	35	F	+	+	+	33,920	new

A = asymptomatic F = fever R = respiratory tract infection.

^1^turned positive with fever following visit.

^2^PCR corrected outcome.

Five children had fever at the time of detection of recurrent infection (Table 2). Four of them had their Giemsa-stained blood smear read on site and were immediately diagnosed and retreated with artemether-lumefantrine. The fifth child did not have the blood smear read on site due to symptoms of respiratory tract infection. This child received antibiotics and improved clinically.

### Sensitivity and specificity

The specificities of HRP2 and LDH against Giemsa-stained blood smear microscopy between days 3 and 42 are shown in Table 3. Compared with PCR the overall (days 0–42) sensitivities and specificities were 68% (95% CI 61–75) and 99% (CI % 98–100) for Giemsa, and 61% (95% CI 53–68) and 98% (95% CI 96–99) for acridine orange-stained blood smear microscopy, respectively. The sensitivity and specificity of acridine orange against Giemsa-stained blood smear microscopy were 81% (95 CI 73–88) and 97% (95% CI 95–98).

**Table 3:** **Specificities of the *Plasmodium falciparum *HRP2 and LDH-based rapid diagnostic tests against Giemsa-stained blood slide microscopy (gold standard) between days 3 and 42**

	**HRP2**			**LDH**			
**Day**		**%**	**CI 95%**		**%**	**CI 95%**	**p-value**
3		NA		20/49	41	27-56	
7		NA		40/53	76	62-86	
14	11/52	21	11-35	51/52	98	90-100	<0.0001
21	17/50	34	21-49	48/50	96	86-100	<0.0001
28	29/49	59	44-73	49/49	100	93-100	<0.0001
35	35/50	70	55-82	48/50	96	86-100	<0.005
42	45/52	87	74-94	50/52	96	87-100	>0.05

NA = not applicable.

## Discussion

The efficiency of two *P. falciparum*-specific RDTs, i.e., HRP2 and LDH were studied for assessment of clearance and detection of recurrent infections during 42 days after initiation of artemether-lumefantrine treatment. This was done through a comparison with two microscopy techniques and real-time PCR. HRP2 had a significantly longer median clearance time (28 days) compared with LDH (seven days). Due to persistent HRP2 positivity from the initial infection, only two out of the ten children with recurrent *P. falciparum* infections during follow-up were identified by HRP2, whereas LDH was able to recognize eight at the time of parasite recurrence. Acridine orange blood smear microscopy did not provide any additional information compared with the other tests used in this study.

### Clearance

Long clearance times for HRP2-based RDTs after ACT treatment have previously been shown [6,26,27], with remaining HRP2 positivity by day 35 in up to 73% of patients despite efficacious treatment. Initial parasite density has been suggested to influence the duration of HRP2 positivity, but a patient’s immune status may also be a contributing factor [28].

Similarly with the present findings, a relatively rapid clearance of LDH has previously been documented [10,12]. It has been claimed that especially HRP2 but also LDH are released by immature gametocytes, which may result in persistent positive test results [5,29]. However, this opinion has been challenged by others who argue that the number of gametocytes after ACT treatment are too few to cause persistent positivity [30]. Importantly, in our this study, only four solely gametocyte carriers were detected by Giemsa-stained blood smear microscopy, of whom three and none were HRP2 and LDH positive, respectively, during gametocyte carriage.

Expert blood smear microscopy remains gold standard for estimation of parasite clearance in clinical trials of anti-malarial drugs. Blood smear positivity by day 3, i.e., 72 hours after initiation of ACT treatment, was observed in 4/53 (8%) of the patients. This is a relatively uncommon finding among African children treated with ACT for uncomplicated malaria [27,31]. The prolonged Giemsa-stained blood smear positivity may be explained by poor compliance since only the initial artemether-lumefantrine dose, i.e., one out of six doses, was given under supervision. Similarly, the relatively long mean PCR clearance time observed may possibly be explained by poor compliance. The presence of gametocyte carriage in patients with prolonged PCR positivity during follow-up may also have influenced the PCR clearance time.

### Parasite density day 0 and persistent HRP2

No correlation was observed between parasite density at enrolment and duration of HRP2 positivity. This is in contrast to several previous publications that report a strong positive correlation [5,6,26,27]. However, these studies have generally included a wider range of parasite densities down to <1,000/μL, where clustering has shown a shorter HRP2 clearance time in parasitaemias <10,000/μL. Conversely, Choidini *et al.* identified a wide range of HRP2 concentrations at equal parasite densities both in panels of cultures and of field isolates [32]. Varying HRP2 concentrations may be dependent on factors such as the duration of infection, the total parasite biomass including the sequestered parasites [33] and anti-HRP2 immune response.

### Detection of recurrent infections

In the moderately high transmission area where the study was conducted, ten (19%) children had recurrent infection detected between days 14 and 35 during follow-up. All recurrent infections were detected by PCR, whereas both the LDH-based RDT and Giemsa-stained blood smear detected eight, HRP2 identified two and acridine orange smear microscopy identified only one of these infections at the time of parasite recurrence. Few other studies have looked at the efficiency of RDTs for detection of recurrent infections during follow-up after anti-malarial treatment. Maxay *et al.* found in a study performed in Thailand that 40/92 (43%) patients experienced recurrent infection during a 28-day follow-up [5]. All these patients had persistent HRP2 positivity from enrolment up to day of recurrent infection.

### RDTs for assessment of treatment outcome

The usefulness of RDTs for monitoring of anti-malarial treatment, i.e., clearance time as well as identification of treatment failure/re-infection, is highly dependent on test specificity. HRP2-based RDTs appear not to be a sufficient tool for this. LDH, with a limited but still longer clearance time as compared with blood smear microscopy, may be useful but for detection of prolonged parasite clearance as a sign of emerging artemisinin tolerance/resistance, it is probably not sufficient.

### RDTs for case detection

Sensitivity and specificity of the HRP2 and LDH antigens for case detection are highly dependent on malaria endemicity [27,28]. In high endemic areas HRP2 generally shows a low specificity, especially among febrile children, because a large proportion of residents have remaining antigenaemia from previous infections. The generally higher parasite densities observed in high endemic areas may also cause longer HRP2 clearance times affecting the specificity. However, Abeku *et al.* showed that in an area of high transmission the specificity increased towards the end of the rainy seasons, and in older age groups probably due to increased HRP2 antibody levels in the population. In low endemic areas the specificity of HRP2 among febrile patients generally increases due to the low malaria incidence and thus the low risk of detecting remaining antigenaemia after cleared infections. LDH, on the other hand, which has shown a comparatively lower sensitivity for detection of *P. falciparum* infections with densities <200/μL, may in areas of low transmission where parasite densities generally are lower especially among asymptomatic cases [34], be a less efficient diagnostic tool than HRP2.

Other limitations with HRP2-based RDTs are the genetic diversity, including the deletions recently described among African *P. falciparum* isolates [35,36], as well as false HRP2 negative results due to the prozone effect [37]. To date, no genetic LDH diversity/deletion has been described and LDH-based RDTs are not susceptible to any prozone effect [38,39]. All these factors should be considered for the choice of RDT in a specific epidemiological setting.

### What does the present study add to the knowledge base of RDTs?

Previous RDT studies in this particular field have mainly followed up the study participants until the RDTs have become negative [12,13]. Conversely, all five diagnostic tests were assessed up to day 42 after treatment initiation in this report. This study design provides a more comprehensive assessment both of clearance times and of recurrent malaria infections for all diagnostic methods, and importantly assesses the efficiency of the two RDTs to detect recurrent malaria infections, which has previously not been reported. Eight of the ten recurrent infections in this study were recognized by LDH at the day of infection and another one on the following sampling day, whereas HRP2 detected only two of the recurrent infections. These results, in combination with the significantly shorter median clearance time of LDH compared with HRP2, provide evidence for the former to be a better tool for monitoring of anti-malarial treatment outcome among children with symptomatic infection in this moderately high endemic area. In addition, this study is the first to incorporate PCR as a comparator for assessment of clearance.

### Limitations

A general limitation when assessing performance of parasite based diagnostic tests that indeed detect different things such as antigens (RDT), DNA (PCR) or whole parasites (microscopy) is that the results are not fully comparable. Furthermore, this is a small study with a limited number of patients with recurrent infections detected during follow-up.

## Conclusion

The LDH-based RDT was superior to HRP2-based for monitoring of treatment outcome and detection of recurrent infections after ACT in this moderately high transmission setting. The results may have implications for the choice of RDT devices in similar transmission settings for improved malaria case management.

## Competing interests

The authors declare that they have no competing interests.

## Authors’ contributions

BAS, MM, AM, AB, and ZP conceived and designed the study. MM, BAS, ZP, and BN carried out and supervised the field work. BAS and UM performed the laboratory analyses. BAS, UM, MM, AB, and AM analysed the data and drafted the manuscript. MP gave important intellectual input in the statistical analysis. All authors read and approved the final manuscript.
